# Impacts of weaning age on dietary needs of whey permeate for pigs at 7 to 11 kg body weight

**DOI:** 10.1186/s40104-021-00637-4

**Published:** 2021-11-16

**Authors:** Ki Beom Jang, Marcos Elias Duarte, Jerry M. Purvis, Sung Woo Kim

**Affiliations:** 1 Department of Animal Science, North Carolina State University, Raleigh, NC 27695 USA; 2 N.G. Purvis Farm Inc, Robbins, NC 27325 USA

**Keywords:** Growth performance, Nursery pigs, Weaning age, Whey permeate

## Abstract

**Background:**

Whey permeate is an effective lactose source for nursery pigs and the most benefits are obtained when pigs are at 7 to 11 kg BW. Altering weaning ages could cause different length of early-weaner phases until 7 kg BW and thus it would influence the dietary need of whey permeate during 7 to 11 kg BW of pigs. This study aimed to evaluate if weaning ages would affect the dietary needs of whey permeate for optimum growth performance of pigs at 7 to 11 kg BW.

**Methods:**

A total of 1,632 pigs were weaned at d 21 (d 21.2 ± 1.3) or d 25 (d 24.6 ± 1.1) after birth. All pigs had a common early-weaner feeds until they reached 7 kg BW. When pigs reached 7 kg BW within a weaning age group, they were allotted in a randomized complete block design (2 × 4 factorial). Two factors were weaning age groups (21 and 25 d of age) and varying whey permeate levels (7.50%, 11.25%, 15.00%, and 18.75%). Data were analyzed using the GLM and NLIN procedures of SAS for slope-ratio and broken-line analyses to determine the growth response to whey permeate and optimal daily whey permeate intake for the growth of the pigs weaned at different ages.

**Results:**

Pigs weaned at 21 d of age had a common diet for 11 d to reach 7 kg BW whereas pigs weaned at 25 d of age needed 2 d. The G:F of pigs weaned at 25 d of age responded to increased daily whey permeate intake greater (*P* < 0.05) than pigs weaned at 21 d of age. Breakpoints were obtained (*P* < 0.05) at 88 and 60 g/d daily whey permeate intake or 17.0% and 14.4% of whey permeate for G:F of pigs weaned at 21 and 25 d of age, respectively.

**Conclusion:**

Pigs weaned at an older age with a short early-weaner phase had a greater growth response to whey permeate intake compared with pigs weaned at a younger age with a long early-weaner phase. Altering weaning ages affected dietary needs of whey permeate for optimum growth performance of pigs from 7 to 11 kg BW.

## Background

Weaning causes morphological, enzymatic, and immunological changes in the gastrointestinal tract of piglets with diet transition from sow milk to plant-based solid feeds [1, 2]. Newly-weaned pigs would be susceptible for diseases due to impaired intestinal barrier function by psychological and environmental stressors, leading to reduction of their growth and survival rate during the post-weaning period [3]. In swine production, weaning age has been generally practiced at range from d 14 to d 28 with most intensive farms having a mean between d 21 and d 28 [4, 5]. Many researchers have studied to evaluate effects of weaning age on growth performance of pigs during the post-weaning period to improve economic efficiency [6–8]. Previous studies have shown that later weaning could improve the wean-to-finish growth rate and intestinal health with reduced mortality during the post-weaning period [7, 8]. Therefore, the efficiency of feedstuffs to provide specific nutrients for the growth of nursery pigs would be altered by the gut maturity based on their weaning ages.

Milk co-products are generally used for nursery pigs to provide lactose as highly digestible energy sources in starter feeds. In particular, whey permeate has the high nutritional value for nursery pigs due to high lactose concentration, which is greatly responsible for the growth response [9–12]. In addition, milk oligosaccharides have been shown to have beneficial impacts on intestinal health of newly weaned pigs [12–14]. Previous studies have shown that lactose, as the primary nutrient in whey permeate positively improved the growth performance of nursery pigs, but the growth responses were gradually disappeared as pigs grew [15–17]. According to our previous finding, the benefits of whey permeate on growth performance were evident during the pre-starter phase which typically includes 7 to 11 kg BW in pig production [12]. Therefore, the pre-starter phase (7 to 11 kg BW) was targeted in this study to evaluate the dietary needs of whey permeate for nursery pigs weaned at different ages.

Based on the previous findings, it is hypothesized that weaning age may alter growth response to whey permeate intake of pigs at 7 to 11 kg BW. To test the hypothesis, the objective of this study was to evaluate if weaning ages (21 or 25 d of age) would affect the dietary needs of whey permeate for optimum growth performance of pigs at 7 to 11 kg BW.

## Materials and methods

The protocol of this experiment was reviewed and approved by North Carolina State University Animal Care and Use Committee (Raleigh, NC). This study was conducted at a commercial swine farm (NG Purvis Farm, Carthage, NC, USA).

### Animals and experimental design

A total of 1,632 pigs with 2 different weaning ages, d 21 (d 21.2 ± 1.3) or d 25 (d 24.6 ± 1.1) after birth, were used in this study. To obtain 1,632 pigs, 163 litters (average parity: 4.3 ± 1.9) were selected based on their farrowing dates. Upon weaning, pigs were moved to a nursery farm and allotted to 48 pens within their weaning age group. Barrows and gilts were mixed in pens. There were 34 pigs per pen needing 1,632 pigs in total. Pigs were fed a common diet until pigs reach 7 kg BW. The common diet consisted of 20.7% crude protein, 13.1% lactose, 8.7% NDF, 0.8% Ca, and 0.7% total P meeting the nutrient requirements suggested by NRC [18]. For the pigs weaned at 21 d of age, a common early-weaner feed was fed for 11 d, whereas for the pigs weaned at 25 d of age, a common early-weaner feed was fed for 2 d. At the end of feeding common diet, the pigs were allotted in a randomized complete block design in a 2 × 4 factorial arrangement, with weaning age groups (21 and 25 d of age) and varying whey permeate levels (7.50%, 11.25%, 15.00%, and 18.75%) as two factors. Each treatment had 6 pens with three BW blocks. After allotment, pigs were provided with treatment feeds and water ad libitum for 10 d to allow reaching 11 kg BW. From d 2 with 2-d intervals, fecal scores of each pen were recorded based on 1 to 5 scale (1: watery and 5: firm) by visual observation of fresh feces. Body weight and feed disappearance were measured at the end of each phase to calculate ADG, ADFI, and G:F.

### Experimental diets

Experimental diets included varying levels of whey permeate (Agri-Mark, Inc., Middlebury, VT) to provide 7.50%, 11.25%, 15.00%, and 18.75% whey permeate (Table 1). Whey permeate was supplemented by altering ratios among corn, crystalline amino acids, dicalcium phosphate, and poultry fat in each diet to match contents of essential nutrients same among treatment diets. All diets were formulated to provide nutrients to meet or exceed requirements suggested by NRC (2012). Feed samples were obtained from each dietary treatment and used to quantify dry matter (DM), gross energy (GE), CP, and lactose. Whey permeate sample was also used to quantify dry matter and lactose.

**Table 1:** Composition of experimental diets

	Whey permeate^a^, %			
Item	7.50	11.25	15.00	18.75
Ingredient, %				
Corn, yellow	53.14	49.39	45.63	41.84
Soybean meal, 48% CP	23.00	23.00	23.00	23.00
Whey permeate	7.50	11.25	15.00	18.75
Blood plasma	3.80	3.80	3.80	3.80
Poultry meal	7.00	7.00	7.00	7.00
Fish meal	2.00	2.00	2.00	2.00
Poultry fat	1.40	1.50	1.60	1.70
Dicalcium phosphate	0.31	0.19	0.08	0.00
Limestone	0.80	0.80	0.80	0.80
L-Lys HCl	0.25	0.26	0.27	0.27
DL-Met	0.11	0.12	0.13	0.14
L-Thr	0.04	0.04	0.04	0.05
Salt	0.22	0.22	0.22	0.22
Vitamin premix^b^	0.03	0.03	0.03	0.03
Trace mineral premix^c^	0.15	0.15	0.15	0.15
Zinc oxide	0.25	0.25	0.25	0.25
Calculated composition				
DM, %	90.2	90.3	90.5	90.6
ME, kcal/kg	3413	3413	3414	3413
CP, %	24.7	24.5	24.3	24.1
SID^d^ Lys, %	1.35	1.35	1.35	1.35
SID Cys + Met, %	0.74	0.74	0.74	0.74
SID Trp, %	0.25	0.25	0.25	0.25
SID Thr, %	0.79	0.79	0.79	0.79
Ca, %	0.80	0.80	0.80	0.81
STTD^e^ P, %	0.40	0.40	0.40	0.40
Total P, %	0.65	0.64	0.63	0.63
Analyzed composition, %				
DM	86.7	86.9	87.5	87.6
CP	24.5	23.7	24.1	23.6
Lactose^f^	5.3	7.9	11.1	13.6

^a^ Treatments were supplemental levels of whey permeate in the diets (Agri-Mark, Inc., Middlebury, Vermont, USA)

^b^ The vitamin premix provided per kilogram of complete diet: 6,614 IU of vitamin A as vitamin A acetate, 992 IU of vitamin D_3_, 19.8 IU of vitamin E, 2.6 mg of vitamin K as menadione sodium bisulfate, 0.03 mg of vitamin B_12_, 4.6 mg of riboflavin, 18.5 mg of D-pantothenic acid as calcium panthonate, 26.5 mg of niacin, and 0.07 mg of biotin

^c^ The trace mineral premix provided per kilogram of complete diet: 33 mg of Mn as manganous oxide, 110 mg of Fe as ferrous sulfate, 110 mg of Zn as zinc sulfate, 16.5 mg of Cu as copper sulfate, 0.3 mg of I as ethylene diamine dihydroiodide, and 0.3 mg of Se as sodium selenite

^d^ *SID* = standardized ileal digestible

^e^ *STTD P* = standardized total tract digestible phosphorus

^f^ Lactose contents were analyzed by University of Missouri AESCL Analytical Services (Columbia, MO)

### Statistical analysis

A randomized complete block design was used in this study with initial BW (heavy, middle, and light) as a blocking criterion. Experimental unit was the pen and data were analyzed using Mixed procedure of SAS 9.4 (SAS Inst. Inc., Cary, NC). Pre-planned orthogonal polynomial contrasts were used to test the effects of weaning age, linear response to supplemental levels of whey permeate, and the interaction between the weaning age and the linear response. Effects of increasing whey permeate levels were also analyzed using the polynomial contrast with coefficients for equally-spaced treatments using Proc IML procedure of SAS 9.4. Growth data was also analyzed using the REG procedure of SAS to evaluate if the growth data was fitted on logarithmic regression.

The comparison of logarithmic regressions between weaning ages was done by the GLM procedure. Logarithmic regressions were obtained between feed efficiency and average daily whey permeate intake to evaluate if the growth response of pigs with increasing daily whey permeate intake was influenced by the weaning age. The data analysis was conducted following procedures described by [19]. The statistic models used in the analysis were as follow: *y* = *a* + *b*_21_ log(*x*_21_) + *b*_25_ log(*x*_25_).

where, *y* = response of growth performance of pigs at 7 to 11 kg BW; *a* is the estimated value of intercept; *x*_25_ and *x*_21_ are the amount of daily whey permeate intake from pigs weaned at 25 or 21 d of age; *b*_25_ and *b*_21_ are the estimated coefficients for the whey permeate intake on each weaning ages when daily whey permeate intake expressed as a logarithmic value. Data were also analyzed using the NLIN procedure of SAS, followed by previous studies [20, 21] for a broken-line analysis to determine an optimal daily whey permeate intake for the growth of pigs. Statistical significance and tendency were considered at *P* < 0.05 and 0.05 ≤ *P* < 0.10, respectively.

## Results

Comparing pigs in different weaning age groups, pigs weaned at 21 d of age had an 11 d early-weaner phase to reach 7 kg BW, whereas pigs weaned at 25 d of age had a 2 d early-weaner phase. Pigs weaned at 21 d of age with an 11 d early-weaner phase to reach 7 kg BW had greater (*P* < 0.05) final BW (from 10.6 to 11.3 kg), ADG (from 347 to 392 g/d), ADFI (from 417 to 517 g/d) and lower feed efficiency (from 0.84 to 0.76) from 7 to 11 kg BW compared with pigs weaned at 25 d of age with a 2 d early-weaner phase (Table 2). Increasing whey permeate levels from 7.50% to 18.75% linearly increased (*P* < 0.05) ADG (from 350 to 391 g/d) and G:F (from 0.77 to 0.83) of pigs without affecting ADFI from 7 to 11 kg BW. The log-linear values in ADG and G:F of pigs from 7 to 11 kg BW were statistically valid (*P* < 0.05). However, There were no interactions between weaning age and linear response to supplemental levels of whey permeate for final BW, ADFI, and G:F of pigs from 7 to 11 kg BW. Fecal score was maintained at 3.4 and was not affected by dietary whey permeate levels regardless of weaning ages of pigs. Mortality of pigs was maintained at 0.04% and was not affected by dietary whey permeate levels regardless of weaning ages of pigs.

**Table 2:** Effects of weaning age on growth performance of nursery pigs fed diets with increasing levels of whey permeate at 7 to 11 kg BW

Weaning age	21 d^a^				25 d^b^					*P* value			
Whey permeate^c^, %	7.50	11.25	15.00	18.75	7.50	11.25	15.00	18.75	SEM	Age	Linear	Log-Linear	Age x Linear
BW, kg													
d 0	7.4	7.4	7.4	7.4	7.1	7.1	7.1	7.1	0.3	0.189	0.989	0.990	0.994
d 10	11.1	11.2	11.3	11.6	10.4	10.5	10.7	10.7	0.4	0.020	0.320	0.329	0.852
ADG, g/d	371	379	394	422	328	342	357	360	15	< 0.001	0.005	0.014	0.567
ADFI, g/d	508	513	515	531	404	415	421	426	16	< 0.001	0.174	0.401	0.994
G:F	0.73	0.74	0.76	0.80	0.81	0.83	0.85	0.85	0.02	< 0.001	0.002	0.014	0.352
Mortality, %	0.0	0.5	0.0	0.5	0.0	1.0	0.5	0.5	0.5	0.425	0.535	0.574	0.999
Fecal score^c^	3.31	3.56	3.66	3.23	3.60	3.39	3.27	3.32	0.2	0.770	0.446	0.763	0.576

^a^Pigs were weaned at 21.2 ± 1.3 d of age and fed a common diet for 11 d until pigs reach 7 kg BW

^b^Pigs were weaned at 24.6 ± 1.1 d of age and fed a common diet for 2 d until pigs reach 7 kg BW

^c^Dietary treatments were supplemental levels of whey permeate in the diets (Agri-Mark, Inc., Middlebury, Vermont, USA)

**Fig. 1:**
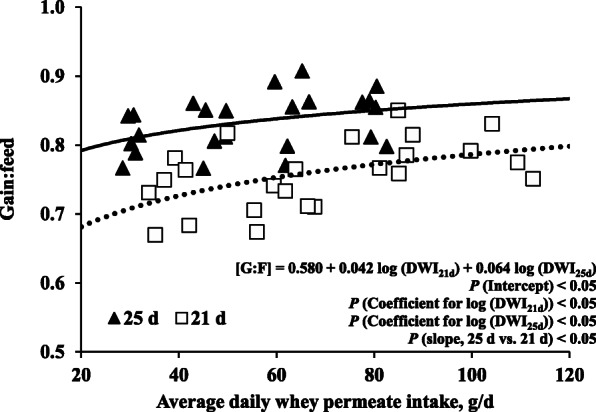
Feed efficiency (G:F) of pigs weaned at different ages (21 and 25 d of age) with increasing average daily whey permeate intake (DWI) from 7 to 11 kg BW. Daily whey permeate intake (g/d) was calculated based on supplemental levels of whey permeate and average daily feed intake. Pigs weaned at 21 and 25 d of age fed a common diet until pigs reach 7 kg BW for 11 and 2 d, respectively. The changes in feed efficiency were: [G:F]_21d_ = 0.487 + 0.065 log (DWI) (*P* < 0.05; R^2^ = 0.24; dotted line) and [G:F]_25d_ = 0.667 + 0.042 log (DWI) (*P* = 0.061; R^2^ = 0.15; solid line). Therefore, [G:F]_overall_ = 0.580 + 0.042 log (DWI_21d_) + 0.064 log (DWI_25d_) (*P* < 0.05; R^2^ = 0.52). The slope-ratio between different weaning ages was different, indicating that the feed efficiency of pigs weaned at 25 d of age responded greater (*P* < 0.05) than growth of pigs weaned at 21 d of age as increasing whey permeate intake

A comparison of the feed efficiency values from the two different weaning ages was shown in Fig. 1. The overall model, intercept, and slopes fitted (*P* < 0.05) G:F as daily whey permeate intake changed. The logarithmic models show that changes of G:F from 7 to 11 kg BW by increasing daily whey permeate were influenced (*P* < 0.05) by weaning ages. The G:F response to daily whey permeate intake of the pigs weaned at 25 d of age with a short early-weaner phase until 7 kg BW was greater than pigs weaned at 21 d of age with a long early-weaner phase.

**Fig. 2:**
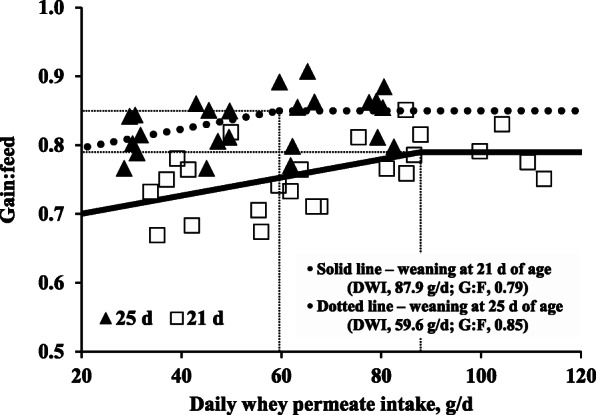
Changes in G:F of nursery pigs at 7 to 11 kg BW by daily whey permeate intake (DWI) using a broken-line analysis (a one-slope model). Daily whey permeate intake (g/d) was calculated based on supplemental levels of whey permeate and average daily feed intake. The breakpoint for G:F of pigs weaned at 21 d of age was 87.9 g/d DWI when their G:F was 0.79 (*P* < 0.05). The breakpoint for G:F of pigs weaned at 25 d of age was 59.6 g/d DWI when their G:F was 0.85 (*P* < 0.05). The statistic models for feed efficiency of weaned pigs were G:F_21_ = 0.79 – 0.13 × 10^− 2^ × (87.9 – DWI) and G:F_25_ = 0.85 – 0.13 × 10^− 2^ × (59.6 – DWI); if DWI is ≥ breakpoint, then DWI = 0

Results using the broken-line analysis revealed that G:F of pigs weaned at 21 d of age was increased (*P* < 0.05) from 0.67 to 0.79 until 87.9 g/d of daily whey permeate intake and then maintained at 0.79, whereas G:F of pigs at 25 d of age was increased (*P* < 0.05) from 0.77 to 0.85 until 59.6 g/d of daily whey permeate intake and then maintained at 0.85 (Fig. 2). Therefore, breakpoints from the broken-line analysis were obtained at 87.9 and 59.6 g/d whey permeate intake for G:F of pigs weaned at 21 and 25 d of age, respectively.

## Discussion

Under a commercial condition, weaning ages can vary among producers depending on preferred management programs. It has been reported that early weaning could potentially result in impaired growth and intestinal health of nursery pigs rather than late weaning [22, 23]. Thus, weaning age could be a factor negatively affecting the nutrient utilization and intestinal barrier functions of nursery pigs [23]. However, there is limited information for nutritional management during post-weaning periods when pigs have different weaning age [18]. In addition, milk coproducts are broadly used in starter feeds as sources of lactose to reduce negative impacts of weaning stress on intestinal health and improve growth performance during the post-weaning period, even though the use of milk coproducts causes economic concerns in swine production. According to our previous finding, feed efficiency of nursery pigs at 7 to 11 kg BW was maximized by daily whey permeate intake at 60 g/d with positive modulation of intestinal microbiota and stimulation of immune response and enterocyte proliferation in the jejunum [12]. Previous studies have suggested various feeding strategies for optimum growth performance of nursery pigs weaned at different ages [24–26]. Jang et al. [12] also showed that supplemental effects of whey permeate on growth performance were most visible at 7 to 11 kg BW that is typically a prestarter phase in pig production. Therefore, the prestarter phase (7 to 11 kg BW) was targeted in this study to evaluate the dietary needs of whey permeate for nursery pigs weaned at different ages. Based on these findings, it was further hypothesized that if altering weaning age causing different length of early-weaner phases until 7 kg BW would affect the growth response and dietary needs of whey permeate for optimal growth performance of pigs at 7 to 11 kg BW.

To the best of our knowledge, this study indicates that early-weaner phase changed the growth response to whey permeate intake and influenced the dietary needs of whey permeate for optimum growth performance of pigs at 7 to 11 kg BW. Interestingly, the pigs weaned at 21 d of age, with early-weaner phase for 11 d until they reached 7 kg BW, had a greater response to whey permeate intake on BW gain and greater dietary needs of whey permeate compared with the pigs weaned at 25 d of age. Therefore, early-weaner phase could be an important factor affecting the growth response to whey permeate intake of pigs at 7 to 11 kg BW. This study also suggests that commercial swine farms may need to consider the inclusion level of whey permeate within their nursery feeding strategies to attain the optimal growth performance of nursery pigs at 7 to 11 kg depending on their weaning age.

The BW gain and feed efficiency of pigs were linearly improved by increasing whey permeate levels at 7 to 11 kg BW. These results were also in accordance with our previous finding that the growth performance of nursery pigs at 7 to 11 kg BW was linearly improved by increasing whey permeate levels [12]. Whey permeate includes lactose as the major component and various milk oligosaccharides as minor components providing functional properties for the intestinal health of nursery pigs [12]. Lactose in milk coproducts would be hydrolyzed to glucose and galactose by lactase secreted from the brush border of small intestine in nursery pigs. Previous studies showed that intestinal lactase activity remains high during nursing period and progressively declined after weaning [27, 28], and thus lactose has been provided as a major energy source in nursery feeds [9–11]. In addition, milk oligosaccharides have been known as bioactive compounds with beneficial effects on growth, immune function, and establishment of intestinal microbiota in neonates [12–14]. Therefore, lactose and milk oligosaccharides in milk coproducts are both beneficial to nursery pigs by enhancing growth and health.

**Fig. 3:**
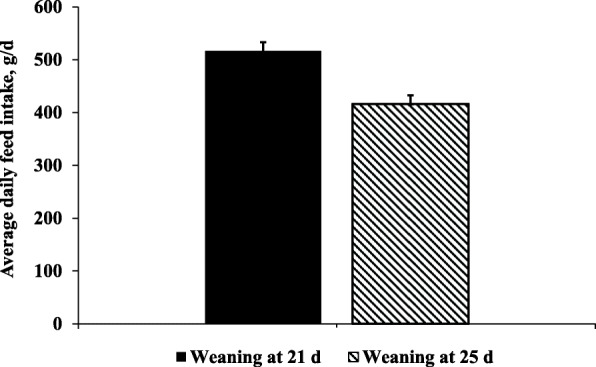
Average daily feed intakes (ADFI, g/d) of the pigs from 7 to 11 kg BW. Pigs were weaned at 21 and 25 d of age fed a common diet until 7 kg for 2 and 11 d, respectively, and fed diets with increasing levels of whey permeate (7.50%, 11.25%, 15.00%, and 18.75%) during 7 to 11 kg BW

This study also shows that weaning age could affect the feed intake as increasing whey permeate levels during 7 to 11 kg BW. According to previous studies, the feed intake and growth would be reduced by weaning stress and impaired intestinal functions of nursery pigs during post-weaning period [29, 30]. In this study, pigs weaned at 25 d of age showed 19% lower feed intake compared with pigs weaned at 21 d of age (Fig. 3). This result could be related to weaning stress in nursery pigs by different length of early-weaner phase until 7 kg BW. According to the previous studies, newly weaned pigs would be exposed to weaning stress leading to damaged intestinal barrier functions with inflammation, and then it could be gradually decreased over the first 2 weeks post-weaning [31, 32]. The early-weaner phase would be required for nursery pigs to reduce the adverse effects of all of the weaning stresses such as environmental, physiological, and social challenges [29]. In this study, the pigs weaned at 25 d of age may have an insufficient early-weaner phase to reduce the negative impacts of weaning stress until reaching 7 kg BW that was 9 d earlier than the pigs weaned at 21 d of age. In addition, according to our previous finding, supplementation of whey permeate positively modulated the intestinal health of nursery pigs through increased enterocyte proliferation and activation of immune response, as well as positive changes in jejunal mucosa-associated microbiota [12]. Therefore, early-weaner phase may affect the response to whey permeate supplementation on feed intake of nursery pigs during 7 to 11 kg BW by reducing the negative impacts from weaning stress and impaired intestinal functions.

Previous studies demonstrated that weaning age could influence growth performance and intestinal function of nursery pigs during post-weaning period [3, 22, 33]. Among various response indicators in slope-ratio analysis, growth performance could be used as a response indicator for availability of nutrients in feed ingredients [34–36]. This study shows that pigs weaned at 25 d of age had a greater response to whey permeate intake on BW gain at 7 to 11 kg BW compared with pigs weaned at 21 d of age. The possible reason is that the early-weaner phase until 7 kg BW may affect the intestinal maturity of nursery pigs. According to Smith et al. [22], the intestinal permeability of nursery pigs was gradually reduced during the first 2 weeks. after weaning. Considering the feeding period from 7 to 11 kg BW, pigs weaned at 25 d of age may have impaired intestinal function with higher intestinal permeability due to a short early-weaner phase compared with pigs weaned at 21 d of age [3, 23]. According to Jang et al. [12], supplementation of whey permeate could stimulate the intestinal development and immune response through increasing the enterocyte proliferation and IL-8 production with positive changes in intestinal microbiota. Thus, whey permeate would be effectively utilized the pigs weaned at old age with a short early-weaner phase to reach 7 kg BW for the growth during 7 to 11 kg BW by improving the intestinal development and maturation of nursery pigs and thus growth response to whey permeate of pigs would be increased to improve the growth and recover from weaning stress during 7 to 11 kg BW.

Feeding the proper amount of lactose would be critical for nursery pigs, because undigested lactose could cause negative impacts on intestinal health including an imbalance of intestinal microbiota and a change of osmotic pressure [37–39]. This study shows that pigs weaned at a younger age require a greater whey permeate intake to have optimum feed efficiency compared with pigs weaned at an older age during 7 to 11 kg BW. Considering their average feed intake in this study, pigs weaned at 21 and 25 d of age may require whey permeate concentration in diets at 17.0% and 14.4%, respectively, to have their optimum feed efficiency during 7 to 11 kg BW. Based on the result in this study, it can be speculated that early-weaner phase could affect dietary whey permeate level to have optimum growth performance of nursery pigs during 7 to 11 kg BW. It has been known that weaning stress could have potential to cause the intestinal dysfunction of nursery pigs during post-weaning period [23]. Li et al. [40] also reported that psychological stresses could cause the reduction of mRNA expression for sodium-glucose transporter (SGLT-1) in the intestine of nursery pigs. Furthermore, previous studies also showed that the intestinal inflammatory responses would be activated during the post-weaning period [29, 41] and it can negatively affect the activities of disaccharidase [42–44]. Therefore, early-weaner phase could influence the dietary needs of whey permeate for optimum growth performance of nursery pigs during 7 to 11 kg BW by intestinal dysfunction induced by weaning stress.

In conclusion, pigs weaned at an older age with a short early-weaner phase until 7 kg BW had a greater growth response to whey permeate intake compared with pigs weaned at a younger age with a sufficient early-weaner phase until 7 kg BW. Altering weaning age causing different length of early-weaner phase until 7 kg BW also affected dietary needs of whey permeate for optimum growth performance of pigs from 7 to 11 kg BW. Optimal levels of whey permeate for growth were 17.0% (or 88 g/d) and 14.4% (or 60 g/d) for pigs from 7 to 11 kg BW when they were weaned at 21 and 25 d of age, respectively. Nursery feeding program should consider post-weaning early-weaner phase to improve the benefits of whey permeate during 7 to 11 kg BW.

## Data Availability

All data generated or analyzed during this study are available from the corresponding author upon reasonable request.

## Abbreviations

ADFI: Average daily feed intake
ADG: Average daily gain
BW: Body weight
CP: Crude protein
DM: Dry matter
DWI: Daily whey permeate intake
Exp: Experiment
G:F: Feed efficiency
GE: Gross energy
mRNA: Messenger ribonucleic acid
NDF: Neutral detergent fiber
SGLT-1: Sodium-glucose transporter
SID: Standardized ileal digestible
STTD P: Standardized total tract digestible phosphorus.
